# Group 2 innate lymphoid cells are numerically and functionally deficient in the triple transgenic mouse model of Alzheimer’s disease

**DOI:** 10.1186/s12974-021-02202-2

**Published:** 2021-07-06

**Authors:** Ivan Ting Hin Fung, Yuanyue Zhang, Damian S. Shin, Poornima Sankar, Xiangwan Sun, Shanti S. D’Souza, Renjie Song, Marcy L. Kuentzel, Sridar V. Chittur, Kristen L. Zuloaga, Qi Yang

**Affiliations:** 1 Department of Immunology and Microbial Disease, Albany Medical College, Albany, NY 12208 USA; 2 Department of Neuroscience and Experimental Therapeutics, Albany Medical College, Albany, NY 12208 USA; 3 Center for Functional Genomics, University at Albany-SUNY, Rensselaer, NY 12144 USA; 4 Biochemistry & Immunology Core Facility at Wadsworth Center, New York State Department of Health, Albany, NY USA

**Keywords:** Alzheimer’s disease (AD), Innate lymphoid cells (ILC), Group 2 innate lymphoid cells (ILC2), IL-5, Cognitive function

## Abstract

**Background:**

The immune pathways in Alzheimer’s disease (AD) remain incompletely understood. Our recent study indicates that tissue-resident group 2 innate lymphoid cells (ILC2) accumulate in the brain barriers of aged mice and that their activation alleviates aging-associated cognitive decline. The regulation and function of ILC2 in AD, however, remain unknown.

**Methods:**

In this study, we examined the numbers and functional capability of ILC2 from the triple transgenic AD mice (3xTg-AD) and control wild-type mice. We investigated the effects of treatment with IL-5, a cytokine produced by ILC2, on the cognitive function of 3xTg-AD mice.

**Results:**

We demonstrate that brain-associated ILC2 are numerically and functionally defective in the triple transgenic AD mouse model (3xTg-AD). The numbers of brain-associated ILC2 were greatly reduced in 7-month-old 3xTg-AD mice of both sexes, compared to those in age- and sex-matched control wild-type mice. The remaining ILC2 in 3xTg-AD mice failed to efficiently produce the type 2 cytokine IL-5 but gained the capability to express a number of proinflammatory genes. Administration of IL-5, a cytokine produced by ILC2, transiently improved spatial recognition and learning in 3xTg-AD mice.

**Conclusion:**

Our results collectively indicate that numerical and functional deficiency of ILC2 might contribute to the cognitive impairment of 3xTg-AD mice.

**Supplementary Information:**

The online version contains supplementary material available at 10.1186/s12974-021-02202-2.

## Introduction

Alzheimer’s disease (AD) is a devastating disease that affects more than 50 million people worldwide. AD is characterized by increased beta-amyloid (Aβ) and tau pathologies and declined cognitive function [1, 2]. The immune system has been implicated as a key factor in AD. Neuroinflammation mediated by dysregulated microglia is a hallmark of AD and is believed to directly contribute to AD pathologies and cognitive decline [3]. Recent studies also indicate potential roles for peripheral and tissue-resident lymphocytes in regulating AD development and progression. CD8^+^ effector memory T cells with enhanced T cell receptor (TCR) signaling accumulated in the brains of AD patients, and their numbers were negatively associated with cognitive function [4]. Depletion of NK cells, the innate lymphocyte subset with cytotoxic function, alleviated neuroinflammation and cognitive decline in the triple transgenic mouse model of AD (3xTg-AD) [5]. Nevertheless, genetic deletion of both adaptive and innate lymphocytes led to exacerbated Aβ pathology in 5xfAD mice, indicating that some other lymphocyte subsets might instead play a protective role in AD [6]. Indeed, enhancing the activity of regulatory T cells (Tregs) improved the cognitive function of APPPS1 mice [7]. However, the function and regulation of other lymphocyte subsets in AD are yet to be better understood.

The 3xTg-AD mice harbor three mutations (*Psen1* PS1M146V, APPSwe, and tauP301L) that lead to Aβ and tau pathologies [8]. 3xTg-AD mice exhibit a progressive age-dependent cognitive decline that varies between sexes [9–12]. Interestingly, substantial changes in immune cell activity have been observed in 3xTg-AD mice [13–15]. Compared to sex- and age-matched control wild-type mice, 3xTg-AD mice exhibit striking splenomegaly, decreased frequency of T lymphocytes, increased anti-dsDNA antibody levels, and dysregulated plasma cytokine concentrations [13–15]. These data together indicate that immunological defects might be closely associated with AD pathologies.

Innate lymphoid cells (ILC) are specialized innate effector cells that lack surface antigen receptors but are functionally analogous to effector T cell subsets [16, 17]. Group 2 innate lymphoid cells (ILC2) express high amounts of GATA3 and produce large amounts of type 2 cytokines IL-5 and IL-13 as well as tissue repair molecules such as Amphiregulin [16, 17]. Our recent work indicates an interesting role for ILC2 and IL-5 in regulating brain function [18]. We found that ILC2 accumulated in the brain barriers such as the choroid plexus (CP) with aging and that their activation alleviated aging-associated cognitive decline via IL-5-dependent mechanisms [18]. The regulation and function of ILC2 in AD, however, remain to be investigated.

In this study, we examined ILC2 phenotype and function in 7-month-old female and male 3xTg-AD mice. Our results indicate remarkable defects in the abundance and functional capability of ILC2 in 3xTg-AD mice of both sexes, compared to age- and sex-matched control wild-type mice. Furthermore, administration of IL-5, a cytokine produced by ILC2, improved spatial learning and recognition in 7-month-old female 3xTg-AD mice. Together, our results reveal a striking deficiency of innate lymphocyte function that might be relevant to the disease outcome in 3xTg-AD mice.

## Methods

### Mice

3xTg-AD and control B6129SF2/J wild-type breeders were obtained from MMRRC JAX [8]. Mice were bred in the animal facility of Albany Medical College. Female and male mice of 7 months old were used in this study. All animal experiments were performed according to the protocols approved by the Institutional Animal Care and Use Committee at Albany Medical College.

### IL-5 treatment

For cytokine treatments, mice were administrated with IL-5 (Biolegend) intraperitoneally. Specifically, mice were treated with 60 μg/kg of IL-5 per dose daily for 2 days on day 1 and day 2; open field, elevated plus maze, and Y-maze forced alternation tests were performed on days 5, 6, and 7 respectively. Mice were treated with 60 μg/kg of IL-5 per dose daily again for 2 days on day 8 and day 9. The water maze test was performed on days 10, 11, 12, and 13. Mice were euthanized on day 14. Thus, each mouse received 4 doses of IL-5 treatment, to a total of 240 μg/kg. Mice were 7 months old at the time of IL-5 treatment and behavior tests.

In some experiments, mice received 4 doses of IL-5 treatment (60 μg/kg per treatment) on day 1, day 2, day 8, and day 9. Mice were rested for 5 weeks after the last IL-5 treatment. Open field (OP), elevated plus maze (EPM), and Y-maze forced alternation task were performed on day 43, day 44, and day 45, respectively. The water maze test was performed on days 48–51. In these experiments, mice were 7 months old at the time of IL-5 treatment and were 8 months old at the time of behavior tests.

### Isolation of hematopoietic cells from whole-brain tissue and from different regions of the brain

For isolation of hematopoietic cells from the whole-brain tissue, mice were perfused through the right ventricle with 50 ml of PBS. The calvaria and dura mater were removed and excluded from the analysis. The whole-brain tissues, including brain parenchyma, choroid plexus, leptomeninges, and perivascular space tissue, were minced with fine scissors and digested in HBSS with 0.2 mg/ml of Liberase TL (Sigma) and 0.1 mg/ml DNase I (Sigma) at 37 °C for 30 min. Cells were filtered through a 70-μm cell strainer. Gradient centrifugation with 40% Percoll (GE) was performed to purify live immune cells. Cells were then stained with various antibodies for flow cytometric analysis or fluorescence-activated cell sorting (FACS).

For isolation of hematopoietic cells from different regions of the brain, the prefrontal cortex (PFC), cortex (CTX), hippocampus (HP), hypothalamus (HT), choroid plexus (CP), perivascular space (PVS), and leptomeningeal (LP) tissues were obtained from mice without perfusion. The tissues were minced with fine scissors and digested in HBSS with 0.2 mg/ml of Liberase TL and 0.1 mg/ml DNase I at 37 °C for 30 min, followed by Percoll gradient centrifugation as described above.

### Flow cytometry and intracellular cytokine staining

Flow cytometry analyses were performed as we previously described [18–23]. Antibodies in the lineage markers included anti-B220 (RA3-6B2), anti-NK1.1 (PK136), anti-CD11b (M1/70), anti-CD3 (2C11), and anti-TCRβ (H57). The lineage antibodies were separated into two channels for a more accurate analysis of ILC2. Specifically, Lin1 (lineage 1) antibodies included FITC-anti-B220 (RA3-6B2), FITC-anti-NK1.1 (PK136), and FITC-anti-CD11b (M1/70). Lin2 (lineage 2) antibodies included PE-anti-CD3 (2C11) and PE-anti-TCRβ (H57). Other antibodies included Pacific Blue-anti-CD45.2 (104), PEcy7-anti-Thy1.2 (53-2.1), Biotin-anti-T1/ST2 (DJ8), SA-PE, APC-anti-IL5 (TRFK5), APC-anti-GATA3 (TWAJ), and PE-anti-CD19 (eBio1D3). Antibodies were purchased from BioLegend, Thermo, or MD Bioscience. DAPI (Thermo) was used to exclude dead cells in the experiments with surface staining only. Flow cytometry analyses were performed with all cells in the liberase-digested whole-brain tissue which contained around 10^5^ immune cells per sample. Single-color controls were used to set up compensations. ILC2 were identified as CD45^+^Lin^−^T1/ST2^+^Thy1.2^+^ cells as we previously described [18–20, 22, 24]. T lymphocytes were identified as CD45^+^CD3^+^ cells. B lymphocytes were identified as CD45^+^CD19^+^ cells. Flow cytometric analysis was performed using a FACSCanto analyzer (BD). Flow cytometry data were acquired by the FACSDiva software (BD), exported as FCS files, and analyzed by the FlowJo software V10 (FlowJo). DAPI was detected by the violet laser of the flow cytometer. FACS sorting was performed using a FACSAria II (BD) three-laser sorter. The area scaling, fluorescence compensation, and sort purity check were performed appropriately for the FACS sorting. For FACS sorting, whole-brain cells from two mice of the same group were pooled into each sample. We pooled whole-brain cells from two mice into one sample, in order to ensure that 100 cells were obtained from each sample. One hundred cells were collected from each sample. Sorting was stopped after 100 cells were collected.

For intracellular staining of GATA3, hematopoietic cells isolated from the whole-brain tissue were first stained with surface markers to identify ILC2 (CD45^+^Lin-T1/ST2^+^Thy1^+^), followed by intracellular staining of GATA3. Intracellular staining of GATA3 was performed using Foxp3 Transcription Factor Staining Buffer Set according to the manufacturer’s instructions (Thermo), as we previously described [18, 19, 24, 25].

Intracellular staining of IL-5 was performed as we previously described [18–27]. Specifically, hematopoietic cells isolated from the whole-brain tissue were re-stimulated with 50 ng/ml PMA and 1 μg/ml ionomycin in the presence of 1 μM monensin at 37 °C for 2.5 h. Cells were then stained with antibodies against surface markers to identify ILC2 (CD45^+^Lin-T1/ST2^+^Thy1^+^), T cells (CD45^+^CD3^+^), or B cells (CD45^+^CD19^+^), followed by intracellular staining of IL-5. For intracellular staining of cytokines from cultured ILC2, cells were incubated with 1 μM monensin in the original culture medium at 37 °C for 2.5 h in the absence of PMA or ionomycin, because the culture medium contained activating cytokines IL-33 and IL-2. Intracellular staining for cytokines was performed using the Cytofix/Cytoperm Kit (BD) according to the manufacturer’s instructions. Live/dead fixable Aqua was used to exclude dead cells in the experiments with intracellular staining.

### ILC2 culture

One hundred ILC2 from the whole-brain tissue were sorted by FACS and cultured in round-bottom 96-well plates in alpha-MEM medium with 20% fetal calf serum (GE, Hyclone Defined) and 1% penicillin-streptomycin, in the presence of 20 ng/ml of IL-7, IL-33, and IL-2. Cells were examined after 7 days of culture by cell counting, flow cytometric analysis, LegendPlex assay, and single-cell RNA-seq (scRNA-seq).

### Measurement of cytokine and β-amyloid concentrations

Cytokine concentrations were measured using the bead-based mouse Th Cytokine Panel (12-plex) LEGENDplex Kit (BioLegend) in a V-bottom plate following the manufacturer’s instructions [28]. The cerebrospinal fluid (CSF) or ILC2 culture supernatant was incubated with a mixture of APC-conjugated LEGENDplex beads at room temperature on a plate shaker for 2 h and washed with washing buffer. The capture beads were conjugated with APC-labeled antibodies that target 12 cytokines. The samples were then labeled with biotin-conjugated detection antibodies for 1 h, and subsequently with streptavidin-conjugated-PE for 30 min. After washing, the samples were examined by flow cytometry analysis. Capture beads that target specific cytokines were differentiated by size and APC fluorescence intensity according to the manufacturer’s instructions. Concentrations of specific cytokines were calculated based on the intensity of PE. Serial-diluted standard controls were used to generate the standard curve.

The concentrations of soluble and insoluble Aβ were measured using the LEGENDMAXTM β-Amyloid x-42 ELISA kit (BioLegend) according to the manufacturer’s instructions. Specifically, brain tissues were homogenized in TBS with 2 mM EDTA and protease inhibitors (Pierce) by a Teflon homogenizer with 5 passes on ice. The tissues were centrifuged for 20 min at 350,000*g*. The supernatant was collected as the soluble fraction. The pellet was resuspended in Triton x-100 in TBS with protease inhibitors and centrifuged again for 20 min at 350,000*g*. After the second round of spinning, the supernatant was collected as the insoluble fraction. Concentrations of Aβ were detected by ELISA using the LEGENDMAXTM β-Amyloid x-42 ELISA kit that was compatible with Triton X-100 non-ionic detergent processing. Due to concerns that behavior tests might alter the Aβ concentrations, Aβ levels were measured in mice that did not undergo behavior tests.

### scRNA-seq

scRNA-seq was performed as we previously described [5, 18, 29]. Specifically, single-cell libraries were generated using Chromium 5” gene expression (10x Genomics) according to the manufacturer’s instructions. Libraries were sequenced by a Next-seq (Illumina) using double-end 75-bp high-throughput sequencing. Initial data analyses were performed with CellRanger v3.1 (10x Genomics) using the default parameters. Data were normalized and scaled using Seurat 3.1.1 [30].. Mitochondrial regression was performed. Principal component analysis and cell clustering were performed using UMAP [31]. Differentially expressed genes were identified by Seurat 3.1.1, with the Wilcoxon rank-sum test used to determine the significance. Gene pathway analyses were performed with DAVID v6.8 [32]. scRNA-seq data were deposited at Gene Expression Omnibus (accession number GSE161861).

### Collection of CSF

CSF was collected from anesthetized mice using a protocol revised from previous work [33]. Specifically, mice were anesthetized by intraperitoneal administration of ketamine (100 mg/kg) and xylazine (20 mg/kg). The hair from between the ears and the neck was removed by Nair. The skin was cleaned with 70% ethanol. A midline incision in the skin was made from the back of the neck to the middle of the skull between the ears. The skin and the connective tissue were pulled apart to expose the cisterna magna. A sharpened glass capillary connected to a microsyringe pump (model DMP, WPI) was aligned against the dura of the cisterna magna. The tip was then punctured into the cisterna magna to collect the CSF. Around 10 to 15 μl of CSF was automatically drawn into the capillary tube and then transferred into a collection tube.

### Immunofluorescence histology

Immunofluorescence histology of amyloid beta was performed with frozen sectioning as previously described [34]. Specifically, mice were sequentially perfused with 50 ml of PBS and 50 ml of 4% paraformaldehyde. The brains were fixed in 4% paraformaldehyde overnight at 4 °C and cryoprotected in 30% sucrose for more than 48 h until tissue sinks at 4 °C. Samples were embedded in an optimal cutting temperature compound (OCT) at − 80 °C. Frozen coronal brain slices were sectioned at 40 μm using a CM1850 cryostat (Leica). Immunofluorescence staining was performed with free-floating sectioning. The sections were incubated with blocking buffer containing using 10% normal goat serum (Jackson ImmunoResearch) in PBS with 0.3% Triton X-100 for 1 h at room temperature and then stained with AF594-conjugated anti-Aβ ab (6E10) (BioLegend, 1:200 dilution) overnight at 4 °C. The sections were mounted onto glass slides, and ProLong Gold Antifade solution (Invitrogen) with DAPI was applied. The slides were imaged using an Axio Observer fluorescence microscope (Zeiss) with a × 5 objective. Images were processed by the Zen Blue software (Zeiss).

### Behavior tests

Mice for behavior tests were habituated to the testing facility for 1 h before starting the behavior tests each day. The tests were recorded and automatically scored by the ANY-maze software (Stoelting Co.). Two independent experiments were performed for each test.

For the open field test, mice explored in a 50 × 50 cm white box for 10 min. Specifically, mice were placed in the southeastern corner of the arena. Mice were allowed to explore the arena for 10 min at which time the mice were video monitored.

For the elevated plus maze test, mice explored in an elevated (39 cm), plus-shaped (+) apparatus with two open and two enclosed arms (5 cm × 33 cm each). Mice were placed at the center of the maze and allowed to explore for 5 min.

For the Y-maze forced alternation test, mice explored a Y-shaped maze (40 cm × 8 cm × 15 cm, L × W × H). Mice were placed at the end of one arm and allowed to explore two arms of the Y-maze for 10 min, with the entry of the third arm blocked. After 1 h, the third arm was open, and mice explored in all three arms for 10 min.

For the water maze test, a circular pool with a diameter of 125 cm was used. The water was made opaque by non-toxic white paint. The temperature was kept at 21–22 °C. Visual cues were placed on four sides of the pool. The maze was conceptually divided into 4 quadrants. On days 1–4, mice were trained to escape the maze by swimming to an invisible plastic platform that was submerged by 1 cm in one of the four virtual quadrants. Mice were placed into the maze from 2 alternating locations opposite to the platform. A trial was concluded when the mouse had found the platform and stayed on it for 10 s. If a mouse failed to escape the maze within 1 min, it was guided slowly to the platform by dragging its tail and stayed on the platform for 10 s. Mice were trained for 4 trials of 1 min each on each day.

### Statistical analysis

For scRNA-seq, the Wilcoxon rank-sum test was used to determine the significance using Seurat V3.1.1. Two-way ANOVA was used to examine the sex difference and water maze scores using Graphpad Prism V7. For other experiments, Student’s *t* tests were used to calculate the statistical significance between the different groups using Graphpad Prism V7. P < 0.05 was considered significant.

## Results

### ILC2 were numerically and functionally compromised in 3xTg-AD mice

**Fig. 1:**
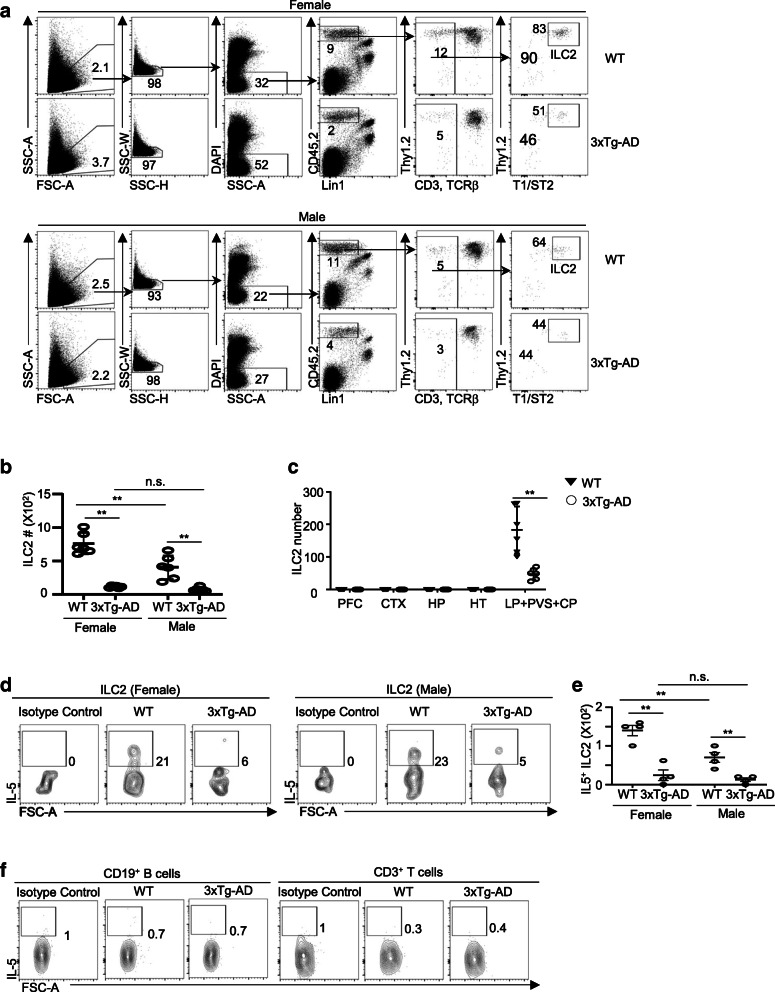
ILC2 are numerically and functionally deficient in 3xTg-AD mice. **a** Representative flow cytometric profiles of ILC2 in the whole-brain tissue of 7-month-old female and male 3xTg-AD mice and control wild-type (WT) mice. ILC2 were identified as Lin^−^CD45^+^Thy1^+^T1/ST2^+^ cells. Lin1 markers included B220, NK1.1, and CD11b. Total Lin markers included B220, NK1.1, CD11b, CD3, and TCRβ. The values shown represent the percentages of gated cells from total cells in the flow cytometry plot. **b** Numbers of ILC2 in the whole-brain tissue of 7-month-old male 3xTg-AD mice and control wild-type mice. Data are from 6 mice per group, two independent experiments. Sex effect: *F* [1, 20] = 15.88, *p* < 0.01; strain effect: *F* [1, 20] = 99.39, *p* < 0.01; interaction: *F* [1, 20] = 10.16, *p* < 0.01. **c** Numbers of ILC2 at different anatomic sites in 7-month-old female and male 3xTg-AD mice and sex and age-matched control wild-type mice. PFC, prefrontal context; CTX, cortex; HP, hippocampus; HT, hypothalamus; LP, leptomeninges; CP, choroid plexus; PVS, perivascular space. Data are from 6 mice per group, two independent experiments. *t* [10] = 4.49*, p* < 0.01. **d** Representative flow cytometric profiles depicting IL-5 expression by ILC2 in the whole-brain tissue of 7-month-old female and male 3xTg-AD and control wild-type mice. Isotype controls were used as the negative control. Plots were pre-gated on ILC2 (Lin^−^CD45^+^Thy1^+^T1/ST2^+^). **e** Numbers of IL-5-expressing ILC2 in the whole-brain tissue of 7-month-old female and male 3xTg-AD and control wild-type mice. Data are from 4 mice per group, two independent experiments. Sex effect: *F* [1, 12] = 12.06, p < 0.01; strain effect: *F* [1, 12] = 54.64, *p* < 0.01; interaction: F [1, 12] = 6.21, *p* < 0.05. **f** Representative flow cytometric profile of IL-5 expression in T cells (CD45^+^CD3^+^) and B cells (CD45^+^CD19^+^) in the whole-brain tissue of 7-month-female and male 3xTg-AD mice and control wild-type mice. Plots were pre-gated on T cells (CD45^+^CD3^+^) or B cells (CD45^+^CD19^+^). Data represent 4 female and 4 male mice per group. Error bars = mean ± SEM. **p < 0.01; n.s., not significant

To understand the regulation and function of ILC2 in AD, we examined ILC2 in 7-month 3xTg-AD mice and control wildtype. This is a time point at which 3xTg-AD mice, particularly female mice, begin to exhibit noticeable declined cognitive function, increased neuroinflammation, and abnormal immunological phenotype [11, 12, 35–38]. We first examined the whole-brain tissue, which contained brain parenchyma, leptomeninges, choroid plexus, and perivascular space tissues, in female and male mice. Notably, both female and male 7-month 3xTg-AD mice exhibit significantly reduced numbers of ILC2 in the brain tissue, compared to age- and sex-matched wild-type mice (Fig. 1A, B). ILC2 were identified as CD45^+^Lin^−^Thy1^+^T1/ST2^+^ cells, as we previously described (Fig. 1A) [19–24, 26, 27, 39]. The remaining ILC2 in 3xTg-AD mice expressed high levels of GATA3, the defining transcriptional regulator of ILC2 (Fig. S1A). The expression of GATA3 and T1/ST2 was comparable between ILC2 in 3xTg-AD mice and those in the control wild-type mice (Fig. S1A, S1B). Our previous work indicated that ILC2 were enriched in the brain barriers such as the choroid plexus tissues of aged C57BL/6 mice [18]. Consistent with the previous results, the brain parenchyma in both wild-type and 3xTg-AD mice were devoid of ILC2 (Fig. 1C). ILC2 were enriched in the barrier tissues including choroid plexus, perivascular tissue, and leptomeninges of wild-type mice, and their numbers were reduced in age- and sex-matched 3xTg-AD mice (Fig. 1C). Thus, ILC2 numbers were reduced in 7-month old 3xTg-AD mice of both sexes.

Previous studies indicate that androgen signaling can repress ILC2 activity [40, 41]. Indeed, we have found that male wild-type mice had fewer numbers of ILC2 in the whole-brain tissue compared to female wild-type mice (Fig. 1B). But no significant difference was observed for the numbers of ILC2 in the whole-brain tissue of female and male 3xTg-AD mice (Fig. 1B). Of note, ILC2 in the lung of male wild-type mice were also fewer than those in female mice, indicating that the sex difference was not specific to the brain-associated tissue (Fig. S1C). However, the number of lung ILC2 was comparable in 3xTg-AD mice and wild-type mice of the same sex (Fig. S1C). Thus, the defects of ILC2 in 3xTg-AD mice are specific to the brain-associated tissue.

To obtain sufficient numbers of ILC2 for more in-depth experiments such as functional assays, we therefore used the whole-brain tissue throughout the rest of the study. Due to their predominant locality in the brain barriers, ILC2 obtained in the whole-brain tissue are referred to as “brain-associated ILC2” in this study. We examined the functional activity of brain-associated ILC2 in 7-month-old female and male 3xTg-AD and control wild-type mice. Our previous study indicates that brain-associated ILC2 in adult C57BL/6 mice produce IL-5 even at homeostasis, although such homeostatic cytokine production declines with age [18]. Consistent with our previous report, brain-associated ILC2 in 7-month-old female and male wild-type mice expressed relatively high amounts of IL-5 (Fig. 1D, E). Notably, the expression of IL-5 was greatly reduced in the remaining brain-associated ILC2 in 3xTg-AD mice, leading to drastically reduced numbers of IL-5-expressing ILC2 in both female and male 3xTg-AD mice (Fig. 1D, E). Together, these data reveal a striking deficiency of functional ILC2 in 3xTg-AD mice.

IL-5 might also be produced by CD3^+^ T cells under certain conditions [42]. However, CD3^+^ T cells or CD19^+^ B cells in the whole-brain tissue did not express significant amounts of IL-5 in 3xTg-AD mice or control wildtype (Fig. 1F). Thus, ILC2 might be the major source of IL-5 in brain-associated tissue.

### ILC2 from 3xTg-AD mice exhibited intrinsic defects in producing IL-5, which was associated with abnormal expression of proinflammatory genes

**Fig. 2:**
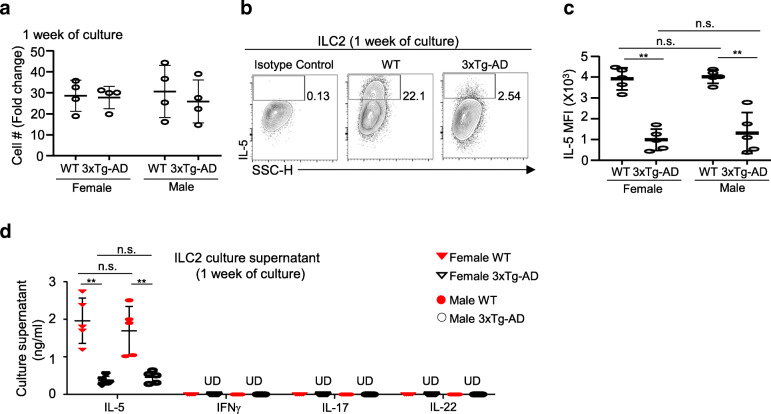
ILC2 from 3xTg-AD mice exhibit intrinsic defects in producing IL-5. **a** 100 ILC2 were purified from the whole-brain tissue of 7-month-old female and male 3xTg-AD mice and control wild-type mice by fluorescence-activated cell sorting (FACS). Specifically, after liberase digestion, whole-brain tissue cells from the two mice of the group were pooled into one sample, and FACS were performed to sort ILC2. We pooled cells from two mice into one sample, in order to ensure that 100 ILC2 were obtained from each sample. One hundred ILC2 were plated into round-bottom 96-well plates and cultured with 20 ng/ml IL-2, IL-7, and IL-33 for 7 days. Growth of ILC2 was calculated as the number of cells that emerged after 7 days of culture per cell plated. Each culture well contained cells pooled from two different mice of the same group. Data are from 4 independent culture well per group, 2 independent experiments. **b** Expression of IL-5 was examined after 7 days of culture. Isotype was used as a negative control. **c** The mean fluorescence intensity (MFI) of IL-5 expression by cultured ILC2, after 7 days of culture. Data are from 5 independent culture well per group, 2 independent experiments. Sex effect: *F* [1, 16] = 0.57, *p* = 0.46; strain effect: *F* [1, 16] = 98.12, *p* < 0.01; interaction: *F* [1, 16] = 0.15, *p* = 0.71. **d** Concentrations of the indicated cytokines in the culture supernatant after 7 days of culture, measured by LegendPLex assays. Data are from 5 independent culture wells per group, 2 independent experiments. Sex effect: *F* [1, 16] = 0.19, *p* = 0.67; strain effect: *F* [1, 16] = 48.83, *p* < 0.01; interaction: *F* [1, 16] = 0.78, *p* = 0.39. U.D., undetected. Error bars = mean ± SEM. **p < 0.01; n.s., not significant

The numerical and functional defects of ILC2 in 3xTg-AD mice could represent transient and reversible changes that can be restored by alteration of environmental factors, or intrinsic irreversible changes such as those imposed by long-term exposure to a destructive microenvironment. To better understand the functional capability of ILC2 from 3xTg-AD mice, we purified ILC2 from 7-month-old 3xTg-AD mice and control wild-type mice by FACS and cultured them in vitro for 7 days with recombinant growth factors IL-2, IL-33, and IL-7. Equivalent numbers of cells from both groups of mice were plated, to avoid potential compounding factors caused by cell clustering effects. Of note, ILC2 from 3xTg-AD mice survived and proliferated in vitro in the presence of exogenous growth factors, at a comparable growth rate as ILC2 from control wild-type mice (Fig. 2A). Nevertheless, ILC2 from 3xTg-AD mice produced significantly lower amounts of IL-5 compared to those from wild-type mice, indicating intrinsic functional defects (Fig. 2B, C). ILC2 from both groups of mice did not produce cytokines belonging to other ILC lymphocytes, such as IFNγ, IL-17, or IL-22, suggesting that they remained relatively committed to the ILC2 lineage (Fig. 2D). Together, ILC2 from 3xTg-AD mice exhibit intrinsic defects in producing cytokines, but not in growth or survival.

Of note, we did not observe a sex difference in cultured ILC2 in both cell growth and cytokine production (Fig. 2A–D). Thus, the sex difference that we observed in vivo was likely due to transient microenvironmental changes such as androgen levels.

**Fig. 3:**
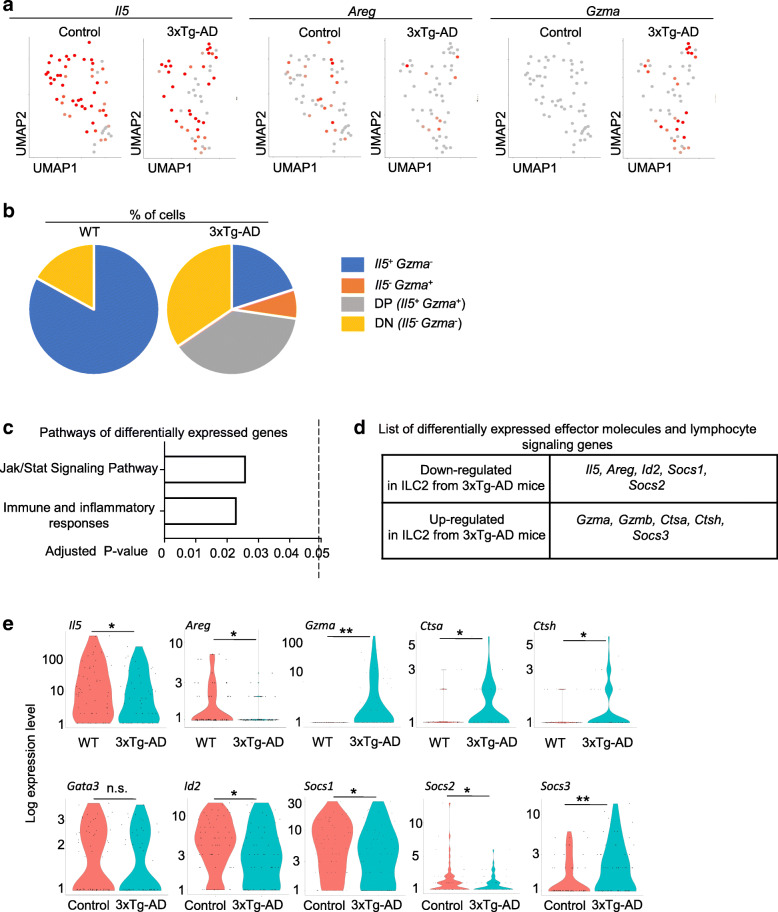
Single-cell transcriptomal profiles of cultured ILC2 from 3xTg-AD mice and control wild-type mice. **a** scRNA-seq was performed with cultured ILC2 from 7-month-old female and male 3xTg-AD mice and age and sex-matched wild-type control mice, after 7 days of culture. Feature plots deposit the expression of the indicated genes. The red color indicates positive expression. The gray color indicates negative expression. **b** Percentages of *Il5*^+^ ILC2, *Gzma*^+^ ILC2, *Il5*^+^ *Gzma*^+^ ILC2 (double-positive), and *Il5*^−^ *Gzma*^−^ ILC2 (double-negative) in cultured ILC2 from 3xTg-AD and wild-type mice, by scRNA-seq. **c** Genes differentially expressed between ILC2 from 3xTg-AD mice and those from control wild-type mice were identified by scRNA-seq. Gene pathway analyses were performed. **d** List of effector molecules and lymphocyte signaling molecules that were differentially expressed between ILC2 from 3xTg-AD mice and those from control wild-type mice. **e** Violin plots depicting the expression of the indicated genes. Cells were pooled from 4 female and 4 male mice per group. *p < 0.05; **p < 0.01; n.s., not significant

To better understand the potential intrinsic defects of ILC2 from 3xTg-AD mice, we performed single-cell RNA-seq with cultured ILC2 from 3xTg-AD and control wild-type mice. As expected, the expression of *Il5* was reduced in ILC2 from 3xTg-AD mice, compared to those from wild-type mice (Fig. 3A). The expression of *Areg*, a tissue repair gene expressed by ILC2, was also significantly reduced in ILC2 from 3xTg-AD mice (Fig. 3A). *Il13* and *Il4* expression were comparable in ILC2 from wild-type and 3xTg-AD mice (Fig. S1D). Notably, ILC2 from 3xTg-AD mice, but not those from control wild-type mice, extensively expressed high amounts of *Gzma* (Fig. 3B). *Gzma* is a cytotoxic molecule involved in the granzyme/perforin cytotoxic pathway, but it also possesses proinflammatory activity independently of its cytotoxic function [43–49]. *Gzma* is traditionally believed to be signature molecules expressed by cytotoxic lymphocytes such as NK/ILC1 and cytotoxic T cells; *Gzma* expression by ILC2 has not been previously reported [50]. Of note, the expression of other genes characteristic of NK/ILC1, such as *Ifng*, *Tbx21*, and *Eomes*, was not detected in cultured ILC2 from 3xTg-AD mice or control wild-type, arguing against the hypothesis that these cells were converted to the ILC1/NK lineage. Because the Seurat software only provides gene expression plots for genes with detectable values, we were not able to provide feature plots or violin plots for these genes (*Ifng*, *Tbx21*, and *Eomes*). We noted that the majority of ILC2 that expressed *Gzma* also expressed high amounts of the *Il5*, the signature effector molecule of the ILC2 lineage (Fig. 3B). Thus, upregulation of *Gzma* likely indicates an increased proinflammatory profile of ILC2 from 3xTg-AD mice, rather than a conversion to a different lineage. Together, ILC2 from 3xTg-AD mice exhibit intrinsic defects in expressing ILC2 characteristic effector molecules *Il5* and *Areg* and gain the capability to co-express proinflammatory molecules that are not regularly expressed by ILC2.

A more comprehensive analysis indicates that the genes differentially expressed between ILC2 from 3xTg-AD mice and those from wild-type control mice were enriched for molecules that participate in immune and inflammatory responses, as well as regulators of lymphocyte activation pathways such as the Jak/Stat pathway (Fig. 3C). In addition to *Gzma*, ILC2 from 3xTg-AD mice also upregulated the expression of *Ctsa* and *Ctsh*, two other cytotoxic molecules with proinflammatory activity (Fig. 3D). In addition, despite comparable expression of *Gata3*, ILC2 from 3xTg-AD mice expressed lower amounts of *Id2*, *Socs1*, and *Socs2*, but higher amounts of *Socs3* (Fig. 3D, E). *Id2* is known to promote ILC development and differentiation [17]. SOCS family members are important regulators of the Jak/Stat pathways and are extensively involved in lymphocyte activation and function [51]. Although the specific role for each SOCS family member in immune cell development and function remains incompletely understood, SOCS1 and SOCS3 appear to play non-redundant roles in regulating immune responses. SOCS1 is essential for CD4^+^ T helper cell differentiation and repression of the expression of cytotoxic molecules including granzymes [52]. SOCS3 may instead play an important role in repressing the signaling pathway of IL-7 [53], an important tropic factor for ILC2. Together, the downregulated expression of *Id2* and *Socs1* and upregulated expression of *Socs3* likely underlie the abnormal functional capability of ILC2 from 3xTg-AD mice.

### Administration of IL-5 improved spatial learning and recognition of 3xTg-AD mice

**Fig. 4:**
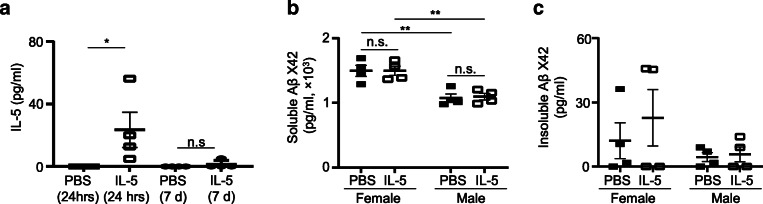
IL-5 treatment did not significantly influence Aβ concentrations in 7-month-old 3xTg-AD mice. **a** Seven-month-old female and male 3xTg-AD mice and sex and age-matched control wild-type mice were treated with IL-5 daily for 2 days. Concentrations of IL-5 in the cerebrospinal fluid (CSF) were examined by LegendPlex assays. Data are from 4 to 6 mice per group, 2 independent experiments. *t* [8] = 2.61, *p* < 0.05. **b** Concentrations of soluble Aβ were examined by ELISA. Data are from 4 mice per group, 2 independent experiments. Sex effect: *F* [1, 12] = 36.05, *p* < 0.01; treatment effect: *F* [1, 12] = 0.029, *p* = 0.87; interaction: *F* [1, 12] = 0.026, *p* = 0.87. **c** Concentrations of insoluble Aβ were examined by ELISA. Data are from 4 mice per group, 2 independent experiments. Error bars = mean ± SEM. *p < 0.05; **p < 0.01; n.s., not significant

To determine whether the defects of ILC2 function in 3xTg-AD mice might be associated with disease outcome, we treated 3xTg-AD mice intraperitoneally (*i.p.*) with IL-5, a cytokine produced by activated ILC2. Mice were treated with 60 μg/kg per dose of IL-5 for 2 days. Our previous work indicates that intraperitoneal injection of an equivalent dose of IL-5 daily for 2 days can improve spatial recognition of aged C57BL/6 mice without noticeable side effects [18]. Systemic administration of IL-5 by intraperitoneal injection led to increased IL-5 levels in the CSF of 7-month-old 3xTg-AD mice, indicating that IL-5 can at least partially cross the blood-brain barrier (Fig. 4A). CSF IL-5 concentrations declined at 7 days post-treatment (Fig. 4A). Acute IL-5 treatment did not significantly affect the levels of soluble or insoluble Aβ in 7-month-old 3xTg-AD mice (Fig. 4B, C). Seven-month-old 3xTg-AD male mice had reduced soluble Aβ concentration in the brain than female mice, but IL-5 treatment did not significantly affect soluble Aβ levels in mice of the same sex (Fig. 4B). Aβ plaques were barely detectable in our 7-month-old 3xTg-AD mice by immunofluorescence staining with the 6E10 antibodies (Fig. S2).

**Fig. 5:**
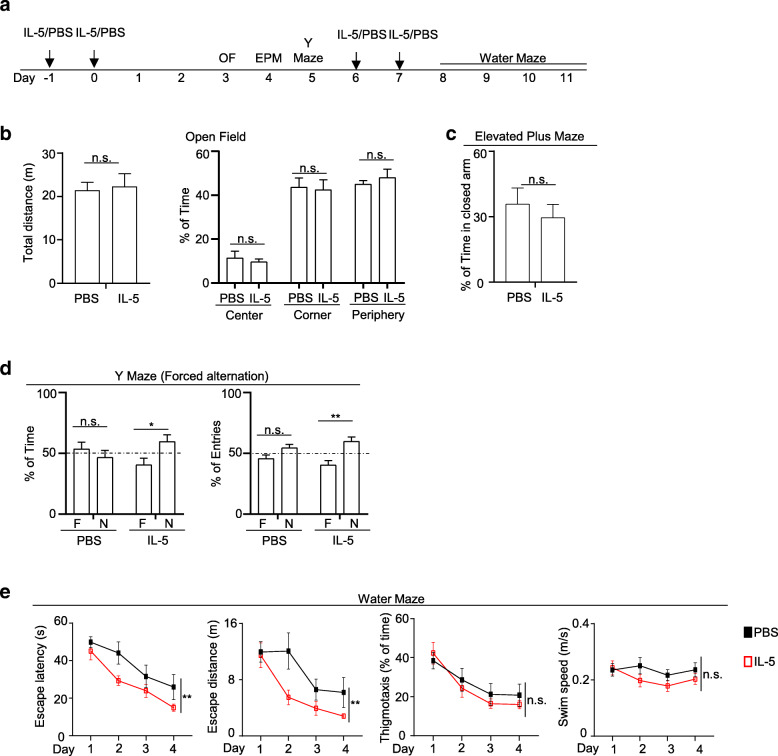
IL-5 treatment improved spatial recognition and learning of 7-month-old female 3xTg-AD mice. **a** Seven-month-old female 3xTg-AD mice were treated with IL-5 and PBS and underwent behavior tests. Experimental scheme for cytokine treatment and behavior tests. OF, open field; EPM, elevated plus maze. **b** Total distance traveled and the percentages of time spent in the indicated zones by open field test. **c** Percentages of time spent in the closed arms in the elevated plus maze test. **d** Percentages of time spent in the novel (N) and familiar (F) arms and percentages of entries into the novel and familiar arms in the Y-maze forced alternation test. Percentage of time: IL-5-treated *t* [18] = 2.42, *p* < 0.05. Percentage of entries: IL-5-treated *t* [18] = 3.7, *p* < 0.01. **e** Escape latency and escape distance to the hidden platform, the thigmotaxis rate, and swim speed during the 4 days of trials in the water maze test. Escape latency: *F*(1, 72) = 8.53, *p* < 0.01. Escape distance: *F*(1, 72) = 8.31, p < 0.01. Data are from 10 mice per group, 2 independent experiments. Error bars = mean ± SEM. *p < 0.05; **p < 0.01; n.s., not significant

We next performed a battery of behavior tests to examine the effects of IL-5 treatment on the cognitive function of 3xTg-AD mice (Fig. 5A). Due to concerns that male 3xTg-AD mice might exhibit unstable behavior phenotype at this age [10, 12], we used 7-month-old female 3xTg-AD mice for all behavior tests in this study. Mice were treated with IL-5 (60 μg/kg per dose) daily for 2 days followed by open field, elevated plus maze, and Y-maze forced alternation tests. Another round of IL-5 treatment (60 μg/kg per dose per day for 2 days) was performed at day 8 and day 9 followed by the water maze test. We performed two rounds of IL-5 treatment, because recombinant IL-5 persisted less than 1 week in the CSF (Fig. 4A). The results from the open field and elevated plus maze tests indicate that IL-5 treatment did not significantly affect the general locomotor or anxiety levels of 3xTg-AD mice (Fig. 5B, C). In the Y-maze forced alternation tests, PBS-treated 7-month-old 3xTg-AD mice failed to recognize the novel arm, demonstrated by an equivalent amount of time spent between the novel arm and the familiar arm (Fig. 5D). Notably, IL-5-treated 3xTg-AD mice spent significantly more time in the novel arm than the familiar arm, suggesting improved spatial recognition (Fig. 5D). We next performed water maze tests to further examine spatial learning. Notably, IL-5-treated 3xTg-AD mice exhibited significantly reduced latency and escape distance to the hidden platform compared to the PBS-treated mice, suggesting improved spatial learning (Fig. 5E). Thigmotaxis rates and swim speed were comparable between IL-5- and PBS-treated 3xTg-AD mice (Fig. 5E). IL-5 treatment did not significantly affect spatial recognition or learning of 7-month-old control wild-type mice (Fig. S3). Together, IL-5 treatment might improve spatial learning and recognition of 7-month-old 3xTg-AD mice.

We next examined whether IL-5 treatment might induce relatively long-term beneficial effects. We treated mice with 4 doses of IL-5 and PBS as described above and let mice rest for 4 weeks. Behavior tests were then performed at 5 weeks after the last IL-5 treatment (Fig. S4A). IL-5-treated mice exhibited similar general behavior and anxiety as PBS-treated mice at 5 weeks post-treatment (Fig. S4B-C). Both groups of mice spent equivalent time in the novel arm and familiar arm in Y-maze forced alternation test (Fig. S4D). In addition, IL-5-treated mice demonstrated generally similar escape latency and escape distance as PBS-treated mice in the water maze test at 5 weeks post-treatment (Fig. S4E). Together, these data indicate that the beneficial effect of IL-5 declines at 5 weeks post-treatment. Thus, the effect of exogenous IL-5 is transient.

## Discussion

In the present work, we have revealed striking numerical and functional defects of ILC2 in 7-month-old 3xTg-AD mice. We demonstrate that ILC2 from 3xTg-AD mice exhibit irreversible defects to produce IL-5 and abnormal expression of proinflammatory genes. We found that treatment with IL-5 may enhance the cognitive function of 3xTg-AD mice. Together, these results indicate that innate lymphocyte defects might be associated with the pathogenesis of AD.

Immunological changes, such as splenomegaly and increased levels of autoimmune antibodies, have been reported in 3xTg-AD mice by previous studies [13–15]. While the previous work explored changes in the peripheral immune system [13–15], our study focused on tissue-resident innate lymphocytes residing at the brain barriers. Potential mechanistic links among the various changes in the peripheral and central nervous system (CNS) immune system in 3xTg-AD mice warrant future investigation. Of note, ILC2 development and function are susceptible to regulation by a variety of systemic or local proinflammatory and anti-inflammatory factors [50]. An altered immunological environment likely underlies ILC2 defects in 3xTg-AD mice. How such changes collectively influence AD development and progression would be an intriguing topic for future investigation. Using in vitro culture experiments, our results indicate and we predict that more extensive analyses of the immunological dysfunction in mouse models of AD and human AD patients will provide important novel insights into the pathogenesis of AD.

Our work indicates that treatment with IL-5, a cytokine produced by ILC2, may enhance spatial recognition of 7-month-old 3xTg-AD mice. Soluble Aβ levels were not significantly altered by IL-5 treatment, and we did not detect significant Aβ plaques at this age. Thus, rather than reducing the Aβ levels, IL-5 might regulate the downstream events induced by Aβ pathologies, such as neuroinflammation or tissue injury, in 7-month-old mice. Indeed, multiple previous studies indicate that ILC2 may play an important role in inflammation resolution and tissue recovery in various lung, gut, and CNS disorders, though the precise mechanisms are yet to be better elucidated [18, 50, 54]. In future studies, it would be interesting to understand whether ILC2 may play a positive role in tissue repair and/or inflammation resolution in neurodegenerative diseases.

This study uses 7-month-old 3xTg-AD mice. Of note, AD predominantly affects the elderly population, and aging can cause drastic changes in the immune system as well as the nervous system [18, 19, 55–61]. In future studies, it will be important to assess the effects of aging on ILC2 homeostasis and function using older AD mice. In addition, substantial sex difference has been observed in 3xTg-AD mice by previous work [10, 12, 13, 38, 62]. In this study, we observed reduced cellularity and function of ILC2 in both female and male 3xTg-AD mice, indicating that ILC2 defects affect 3xTg-AD mice of both sexes. Nevertheless, we only performed behavior tests in female 3xTg-AD mice, due to concerns that male mice at this age might exhibit unstable behavior phenotype [10, 12]. Future studies to investigate whether enhancing ILC2 function can improve cognitive function in male 3xTg-AD mice, particularly those at an older age, would be highly worthwhile.

## Conclusion

In conclusion, we report that ILC2 are numerically and functionally deficient in 3xTg-AD mice. In addition, treatment with IL-5, a cytokine produced by ILC2, transiently enhances the cognitive function of 3xTg-AD mice. Together, these results indicate that numerical and functional deficits of ILC2 might contribute to the cognitive impairment of 3xTg-AD mice.

## Supplementary Information

**Additional file 1: Fig. S1.** Characterization of ILC2 in 7-month old 3xTg-AD and control wild-type mice. (**A**) Representative flow cytometric profiles depicting GATA3 and T1/ST2 expression in ILC2 from the whole brain tissue of 7-month old female and male 3xTg-AD mice and age and sex-matched control wildtype mice. Plots were pre-gated on ILC2 (Lin^-^CD45^+^Thy1^+^T1/ST2 (**B**) Mean fluorescence intensity (MFI) of GATA3 and T1/ST2 expression in ILC2 from the whole brain tissue of 7-month old female and male 3xTg-AD mice and sex and age-matched control wildtype mice. Data are from 6 mice per group, two independent experiments. (**C**) Numbers of ILC2 in the lungs of 7-month old female and male 3xTg-AD mice and control wildtype mice. Data are from 4 mice per group, 2 independent experiments. Sex effect: *F* [1, 12] = 42.49, *p*<0.01; Strain effect: *F* [1, 12] = 0.23, *p* = 0.64; Interaction: *F* [1, 12] = 0.23, *p* = 0.64. (**D**) scRNA-seq was performed with cultured ILC2 from 7-month old female and male 3xTg-AD mice and age and sex-matched wildtype control mice, after 7 days of culture. Violin plots depict the expression of the indicated genes. Error bars = mean ± SEM. **p<0.01; n.s., not significant. **Fig. S2.** Immunofluorescence staining of 7-month old 3xTg-AD mice treated with PBS or IL-5. 7-month old female and male 3xTg-AD mice were treated with IL-5 or PBS daily for 2 days. Brain sections were obtained at 24 hours after treatment and stained with 6E10 antibodies. Representative immunofluorescence images were shown. Brain sections of 12-month old female 3xTg-AD mice were used as positive controls. Data represent 4 female and male mice per group, two independent experiments. **Fig. S3.** Behavior test results of 7-month old female wildtype control mice that were treated with IL-5 or PBS control. 7-month old female wildtype control mice were treated with IL-5 and PBS, and underwent behavior tests as described in Fig. 5A. (**A**) The Open Field arena was virtually divided into “Center”, “Periphery”, and “Corner” zones in Open Field test. (**B**) Concentrations of IL-5 in the cerebrospinal fluid (CSF) were examined in 7-month old female wildtype mice, at 24 hours after treatment with IL-5 or PBS daily for 2 days. Data are from 6 mice per group, two independent experiments. (**C**) Total distance traveled, and percentages of time spent in the indicated zones in the Open Field test. (**D**) Percentages of time spent in the closed arms in the Elevated Plus Maze test. (**E**) Percentages of time spent in the novel (N) and familiar (F) arms, and the percentages of entries into the novel and familiar arms, in the Y-maze forced alternation test. Percentage of time: PBS-treated *t* [18] = 5.47, *p*<0.01, IL-5-treated *t* [18] = 5.86, *p*<0.01. Percentage of entries: PBS-treated *t* [18] = 7.1, *p*<0.01, IL-5-treated *t* [18] = 3.66, p<0.01. (**F**) Escape latency and escape distance to the hidden platform, and the thigmotaxis rate during the 4 days of trials in the Water Maze test. Data are from 10 mice per group (C-F), 2 independent experiments. Error bars = mean ± SEM. **p<0.01; n.s., not significant. **Fig. S4.** Behavior test results of 7-month old 3xTG-AD mice 4 weeks after treatment with IL-5 or PBS control. 7-month old female 3xTG-AD mice were treated with IL-5 and PBS and were rested for 5 weeks, then underwent behavior tests. (**A**) Experimental scheme for cytokine treatment and behavior tests. OF, Open Field; EPM, Elevated Plus Maze. (**B**) Total distance traveled, and the percentages of time spent in the indicated zones by Open Field test. (**C**) Percentages of time spent in the closed arms in the Elevated Plus Maze test. (**D**) Percentages of time spent in the novel (N) and familiar (F) arms, and the percentages of entries into the novel and familiar arms, in the Y-maze forced alternation test. (**E**) Escape latency and escape distance to the hidden platform, and the thigmotaxis rate during the 4 days of trials in the Water Maze test. Data are from 10 mice per group, 2 independent experiments. Error bars = mean ± SEM; n.s., not significant.

## Data Availability

scRNA-seq data were deposited at the Gene Expression Omnibus (accession number GSE161861).

## Abbreviations

Aβ: Beta-amyloid
ILC: Innate lymphoid cells
ILC2: Group 2 innate lymphoid cells
*i.p.*: Intraperitoneal
Tregs: Regulatory T cells
AD: Alzheimer’s disease
TCR: T cell receptor
WT: Wild-type
MFI: Mean fluorescence intensity
PFC: Prefrontal cortex
CTX: Cortex
HP: Hippocampus
HT: Hypothalamus
LP: Leptomeninge
PVS: Perivascular space
CP: Choroid plexus
CNS: Central nervous system
