# Supplementary figures and images for: Group 2 innate lymphoid cells are numerically and functionally deficient in the triple transgenic mouse model of Alzheimer’s disease
Source: J Neuroinflammation. 2021 Jul 6;18:152. doi: 10.1186/s12974-021-02202-2 (PMC8261980; doi:10.1186/s12974-021-02202-2)

### Supplementary Figure S1

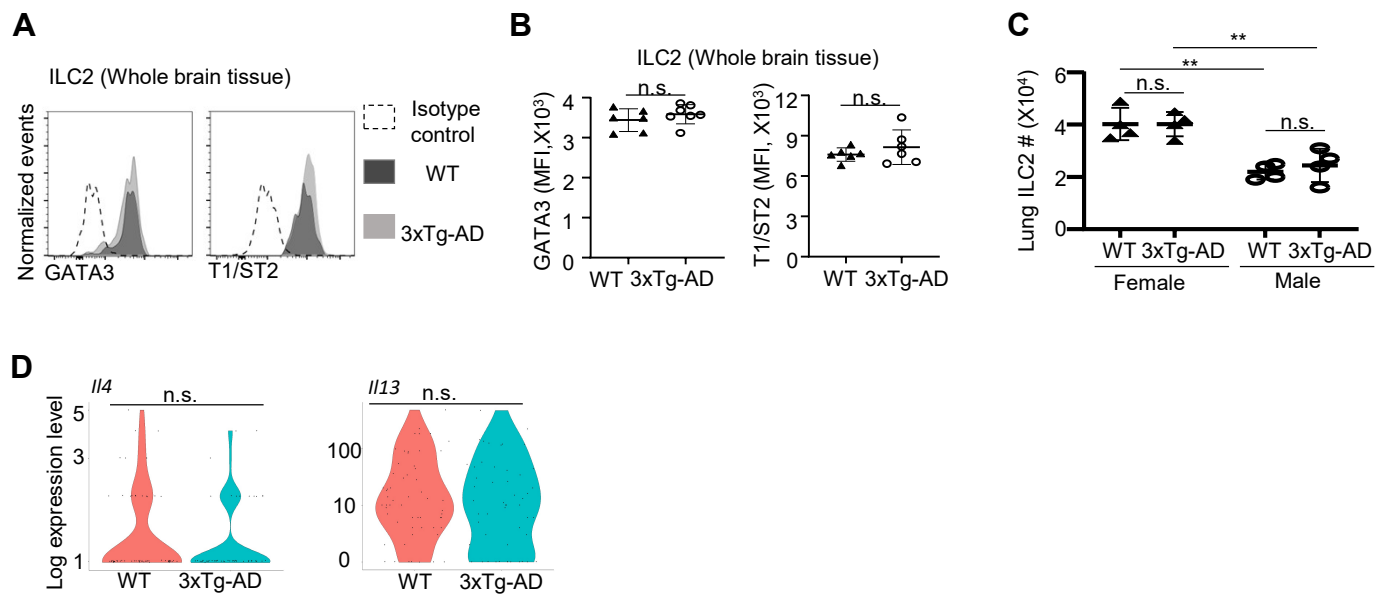

Supplementary Figure S2

A

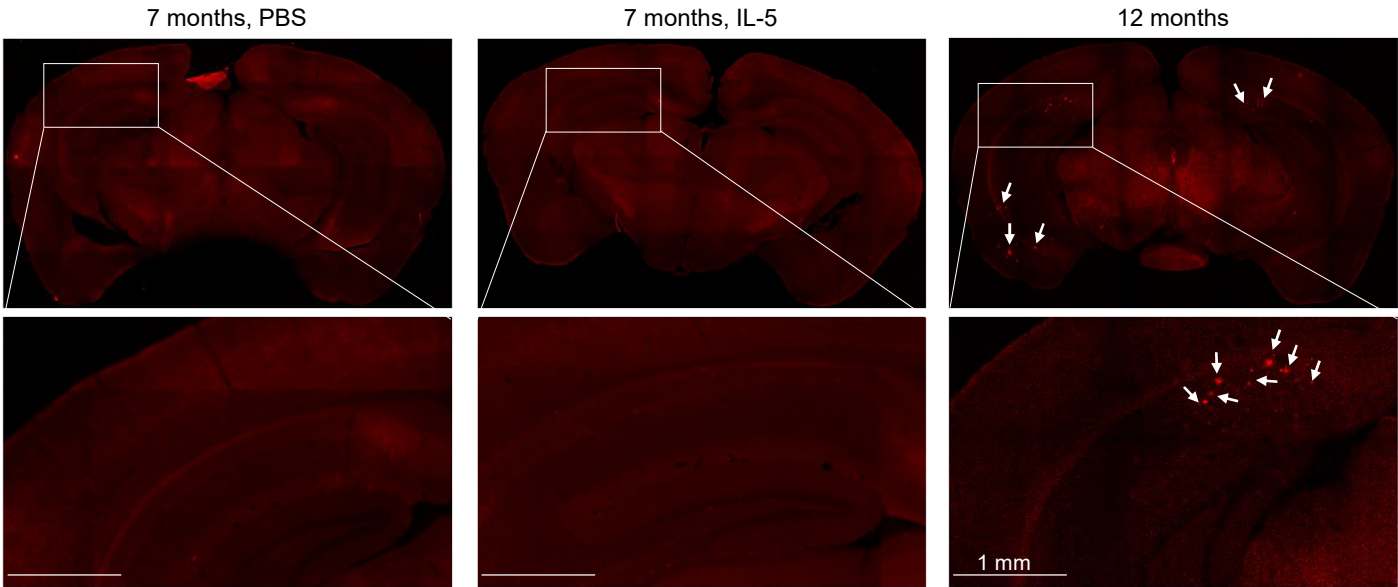

Supplementary Figure S3

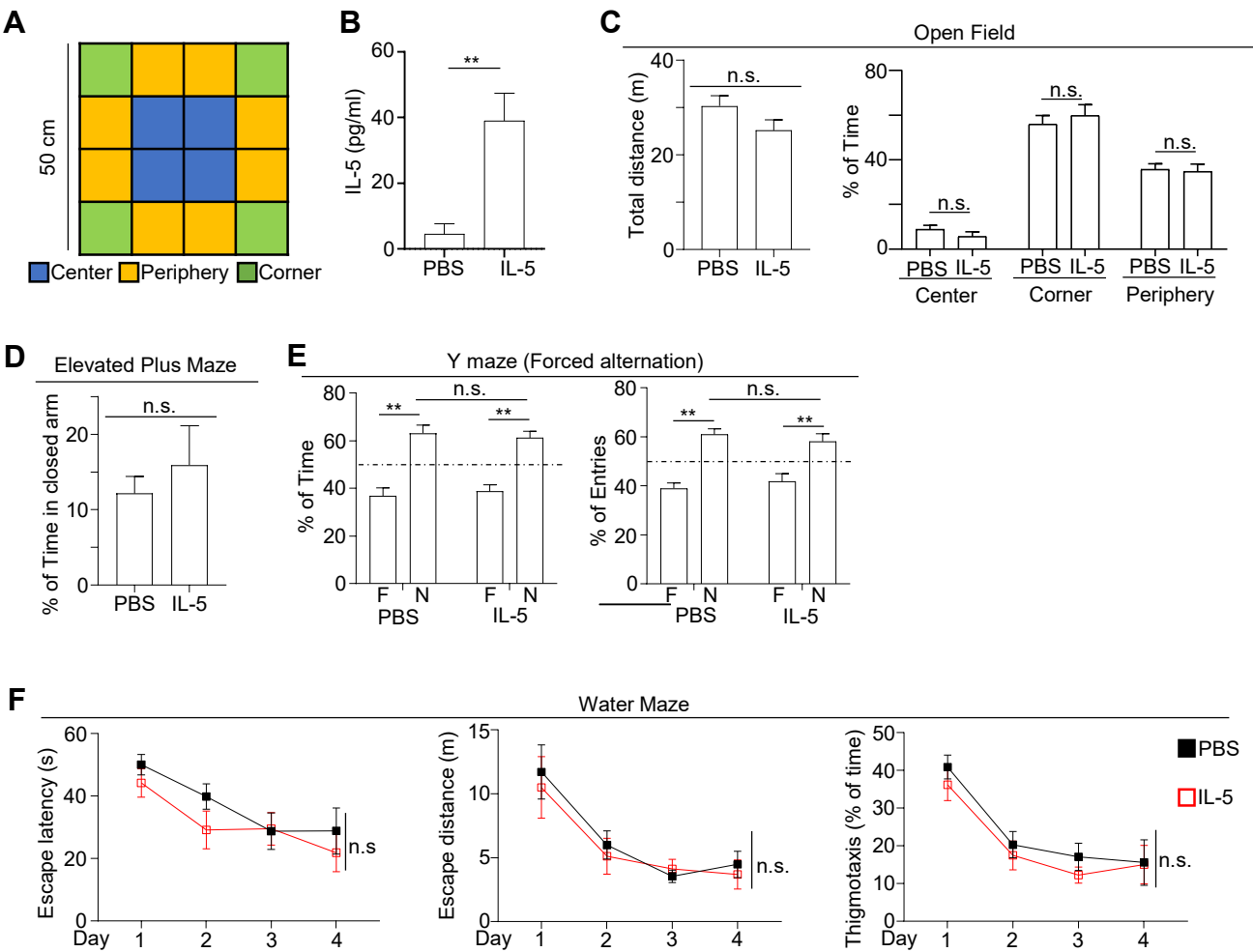

**Fig. S4**

**A**

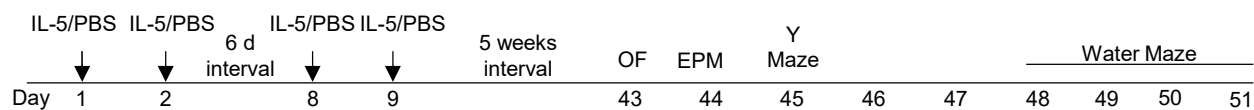

**B**

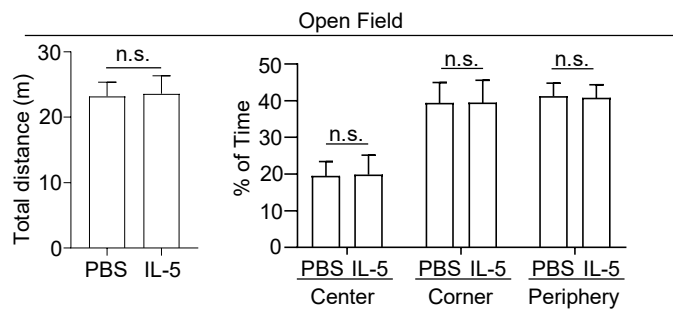

**C**

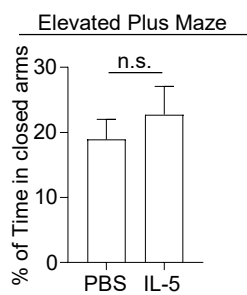

**D**

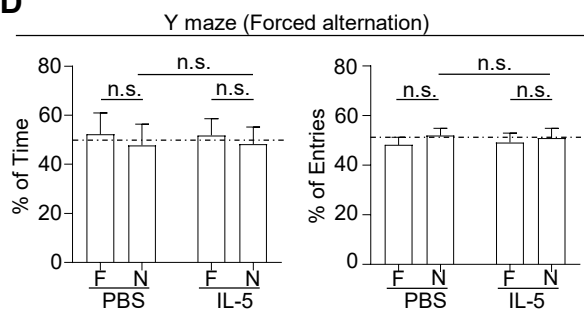

**E**

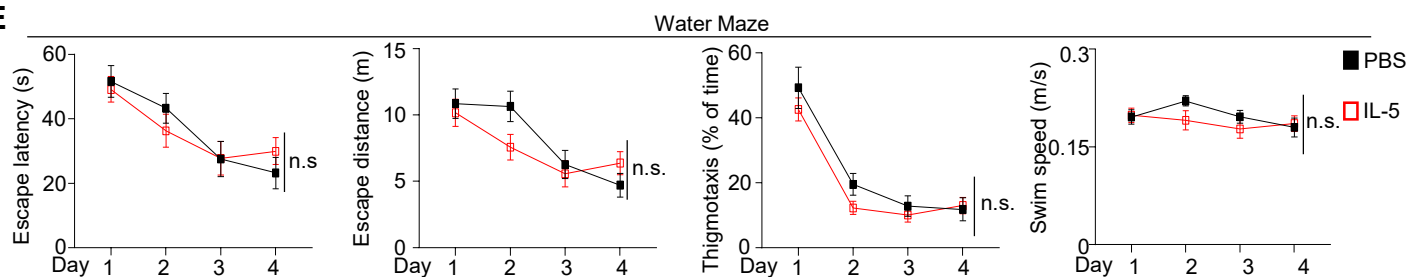

Supplement: Supplementary file 1 — Additional file 1: Fig. S1. Characterization of ILC2 in 7-month old 3xTg-AD and control wild-type mice. (A) Representative flow cytometric profiles depicting GATA3 and T1/ST2 expression in ILC2 from the whole brain tissue of 7-month old female and male 3xTg-AD mice and age and sex-matched control wildtype mice. Plots were pre-gated on ILC2 (Lin-CD45+Thy1+T1/ST2 (B) Mean fluorescence intensity (MFI) of GATA3 and T1/ST2 expression in ILC2 from the whole brain tissue of 7-month old female and male 3xTg-AD mice and sex and age-matched control wildtype mice. Data are from 6 mice per group, two independent experiments. (C) Numbers of ILC2 in the lungs of 7-month old female and male 3xTg-AD mice and control wildtype mice. Data are from 4 mice per group, 2 independent experiments. Sex effect: F [1, 12] = 42.49, p<0.01; Strain effect: F [1, 12] = 0.23, p = 0.64; Interaction: F [1, 12] = 0.23, p = 0.64. (D) scRNA-seq was performed with cultured ILC2 from 7-month old female and male 3xTg-AD mice and age and sex-matched wildtype control mice, after 7 days of culture. Violin plots depict the expression of the indicated genes. Error bars = mean ± SEM. **p<0.01; n.s., not significant. Fig. S2. Immunofluorescence staining of 7-month old 3xTg-AD mice treated with PBS or IL-5. 7-month old female and male 3xTg-AD mice were treated with IL-5 or PBS daily for 2 days. Brain sections were obtained at 24 hours after treatment and stained with 6E10 antibodies. Representative immunofluorescence images were shown. Brain sections of 12-month old female 3xTg-AD mice were used as positive controls. Data represent 4 female and male mice per group, two independent experiments. Fig. S3. Behavior test results of 7-month old female wildtype control mice that were treated with IL-5 or PBS control. 7-month old female wildtype control mice were treated with IL-5 and PBS, and underwent behavior tests as described in Fig. 5A. (A) The Open Field arena was virtually divided into “Center”, “Periphery”, and “ [file 12974_2021_2202_MOESM1_ESM.pdf]
